# Aging-induced YTHDF aggregates impair mitochondrial function by trapping mitochondrial RNAs and suppressing their expression in the brain

**DOI:** 10.1093/procel/pwad041

**Published:** 2023-07-04

**Authors:** Juan Zhang, Dingfeng Li, Keqiang He, Qiang Liu, Zhongwen Xie

**Affiliations:** School of Life Sciences, Anhui Agricultural University, Hefei 230036, China; State Key Laboratory of Tea Plant Biology and Utilization, School of Tea and Food Sciences and Technology, Anhui Agricultural University, Hefei 230036, China; Division of Life Sciences and Medicine, Hefei National Research Center for Physical Sciences at the Microscale, University of Science and Technology of China, Hefei 230026, China; Department of Anesthesiology, The First Affiliated Hospital of USTC, Division of Life Sciences and Medicine, University of Science and Technology of China, Hefei 230001, China; Division of Life Sciences and Medicine, Hefei National Research Center for Physical Sciences at the Microscale, University of Science and Technology of China, Hefei 230026, China; Anhui Province Key Laboratory of Biomedical Aging Research, University of Science and Technology of China, Hefei 230026, China; Center for Advanced Interdisciplinary Science and Biomedicine of IHM, Division of Life Sciences and Medicine, University of Science and Technology of China, Hefei 230026, China; State Key Laboratory of Tea Plant Biology and Utilization, School of Tea and Food Sciences and Technology, Anhui Agricultural University, Hefei 230036, China

## Dear Editor,

Brain aging is associated with a decrease in cognitive function, which is often accompanied by defective changes in mitochondrial morphology and reduction in mitochondrial function (Faitg et al., 2021). The main function of mitochondria is to produce energy in the form of ATP, which is vital to fulfill high energy demand of neurons, mainly for synaptic processes (Lee et al., 2018). Mitochondrial dysfunction acts as a key factor for age-related cognitive decline (Grimm and Eckert, 2017).

Aging induces the formation of insoluble protein aggregates in the brain (Kelmer et al., 2020). YTHDF proteins contain low-complexity domain (LCD) and have potential to form protein aggregates through phase–phase separation under stress conditions (Ries et al., 2019). The YTHDF proteins are ubiquitously expressed in eukaryotes and are known to regulate gene expression through modulating RNA metabolism under physiological condition (Wang et al., 2014; Zaccara and Jaffrey, 2020). *Ythdf2* knockout mice are embryonic lethal and *Ythdf1* knockout mice exhibit memory defects (Shi et al., 2018; Zhao et al., 2017). Aging is considered a chronic stress, we thus examined the aggregation properties of YTHDF proteins during brain aging and investigated their contribution to brain aging.

**Figure 1.**
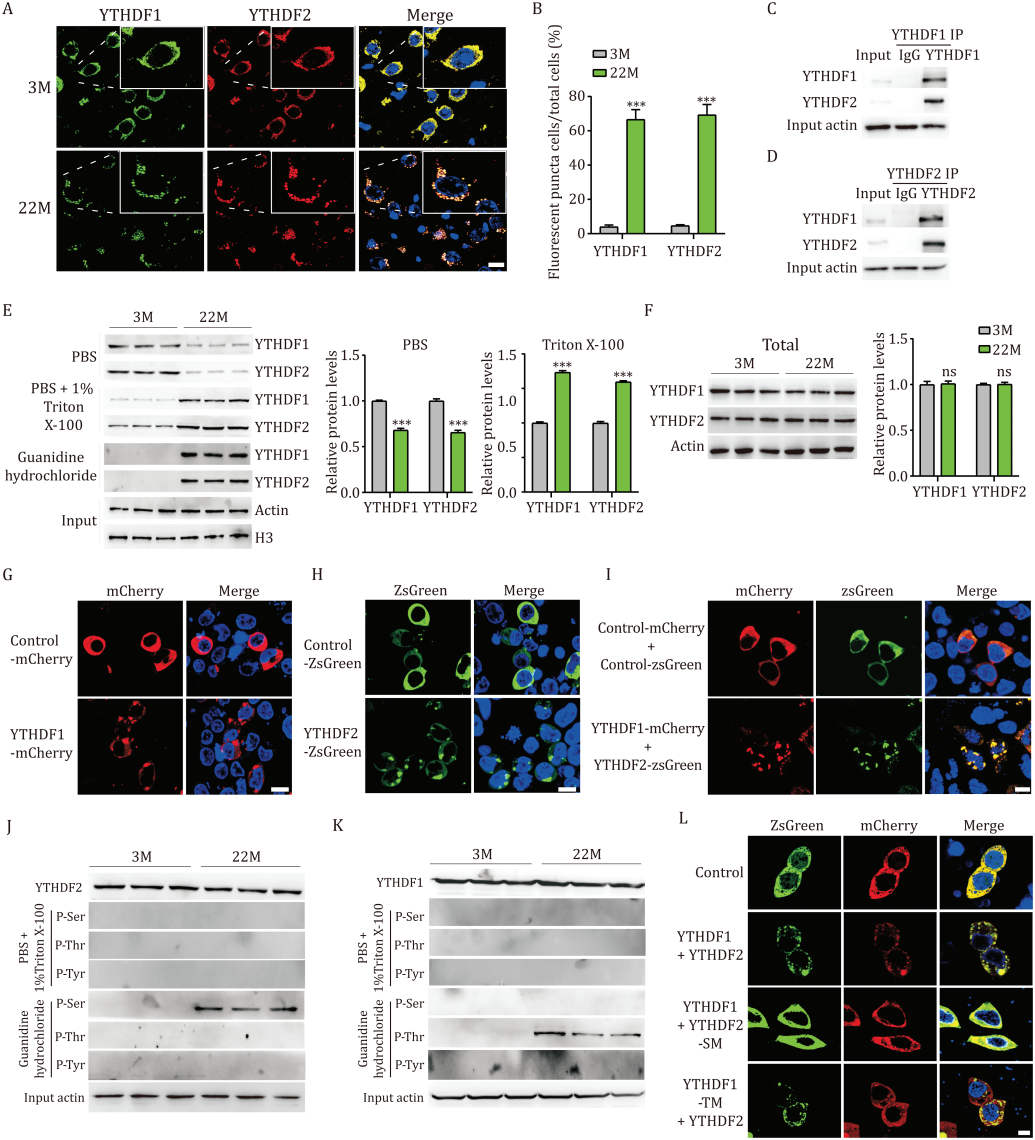
**YTHDF proteins form detergent-insoluble aggregates during brain aging and this formation is dependent on their phosphorylation modifications.** (A) Representative immunofluorescence images of YTHDF1 (green), YTHDF2 (red), and DAPI (blue) in the mPFCs of 3-month (3M) and 22-month (22M) mice. Scale bar: 20 µm. (B) The percentage of cells with YTHDF1 or YTHDF2 fluorescent puncta in the mPFCs of 3M vs. 22M mice (*n* = 100). (C and D) mPFC homogenates from 22M were subjected to immunoprecipitation with an anti-YTHDF1 antibody (C) or an anti-YTHDF2 antibody (D), followed by detection with anti-YTHDF1 and anti-YTHDF2 antibodies. (E) 3M and 22M mPFCs were sequentially extracted by PBS, PBS + 1%Triton X-100, and 5M guanidine hydrochloride. Levels of YTHDF1 and YTHDF2 proteins in each extract were determined by immunoblotting and densiometric analysis (*n* = 3). Levels of actin and H3 were included as input controls. (F) Levels of YTHDF1 and YTHDF2 proteins in 5M guanidine hydrochloride extracted mPFCs of 3M and 22M mice, by immunoblotting and densiometric analysis (*n* = 3). Actin was included as an input control. (G) Representative fluorescence images of YTHDF1 (red) and DAPI (blue) in N2a cells transfected with control-mCherry or YTHDF1-mCherry plasmids. Scale bar: 20 µm. (H) Representative fluorescence images of YTHDF2 (green) and DAPI (blue) in N2a cells transfected with control-ZsGreen or YTHDF2-ZsGreen plasmids. Scale bar: 20 µm. (I) Representative fluorescence images of YTHDF1 (red), YTHDF2 (green), and DAPI (blue) in N2a cells transfected with control or YTHDF1-mCherry + YTHDF2-ZsGreenplasmids. Scale bar: 20 µm. (J and K) Total levels of YTHDF1 and YTHDF2 proteins in 5M guanidine hydrochloride extracted 3M and 22M mPFCs. 3M and 22M mPFCs were sequentially extracted by PBS + 1% Triton X-100 and 5M guanidine hydrochloride, and each extract was subjected to immunoprecipitation with an anti-YTHDF2 antibody (J) or an anti-YTHDF1 antibody (K), followed by immunoblotting with anti-p-Ser, anti-p-Thr, and anti-p-Tyr antibodies. (L) Representative fluorescence images of YTHDF1 (red), YTHDF2 (green), and DAPI (blue) in N2a cells transfected with control, YTHDF1-mCherry + YTHDF2-zsGreen, or YTHDF1-mCherry + YTHDF2-SM-zsGreen, or YTHDF1-TM-mCherry + YTHDF2-zsGreen plasmids. Scale bar: 10 µm. For this and subsequent figures 3M and 22M represent 3-month-old and 22-month-old mice. ns, not significant; ^***^P < 0.001 by ANOVA or Student’s *t*-test; error bars denote the SEM.

To investigate whether aging could induce the formation of YTHDF aggregates in aged brains, we conducted immunofluorescence staining and observed that both YTHDF1 and YTHDF2 formed visible cytoplasmic aggregates in the mPFCs of 22-month-old (22M) mice, whereas no obvious aggregates were detected in the mPFCs of 3-month-old (3M) mice (Fig. 1A and 1B). Moreover, these cytoplasmic aggregates in the mPFCs of 22M were both YTHDF1 and YTHDF2 positive (Fig. 1A), suggesting that YTHDF1 and YTHDF2 form co-aggregates in aged brains. These findings suggest that formation of YTHDF1 and YTHDF2 aggregates and their interaction are associated with brain aging. In support of this, we detected an association of YTHDF1 and YTHDF2 in the mPFCs of 22M mice by conducting co-immunoprecipitation (Co-IP) (Fig. 1C and 1D). To identify the interacting domain of YTHDF1 and YTHDF2, we next generated truncated fragments of YTHDF proteins: N-terminal fragments (YTHDF1-M1 and YTHDF2-M1) and C-terminal fragments (YTHDF1-M2 and YTHDF2-M2) (Fig. S1A and S1B). We detected an association of YTHDF1-M1 and YTHDF2, and detected no association of YTHDF1-M2 and YTHDF2, suggesting that YTHDF1 interacts with YTHDF2 through its N-terminal domain (Fig. S1C). Similarly, we detected an association of YTHDF2-M1 and YTHDF1, and detected no association of YTHDF2-M2 and YTHDF1, suggesting that YTHDF2 interacts with YTHDF1 through its N-terminal domain (Fig. S1D). By generating additional truncated fragments on the N-terminus (YTHDF1-M3, YTHDF1-M4, YTHDF2-M3, YTHDF2-M4), we confirmed that Pro/Gln rich domain on the N-terminus is required for YTHDF1–YTHDF2 interaction (Fig. S1C and S1D). These findings suggest that aging induces formation of cytoplasmic YTHDF1 and YTHDF2 positive aggregates and YTHDF1–YTHDF2 interaction depends on their Pro/Gln rich domains.

To determine the solubility of YTHDF aggregates, we generated solubility profiles by applying a sequential extraction approach on 22M and 3M mPFCs using buffers of increasing stringency (Youmans et al., 2011). mPFCs were sequentially extracted by PBS (soluble fraction) and 1% Triton X-100 in PBS (detergent soluble fraction). Triton-resistant fraction (insoluble aggregates) was solubilized with 5M guanidine hydrochloride. By conducting immunoblotting for each extraction, we observed that YTHDF2 was significantly reduced in PBS extraction of 22M mPFCs and significantly increased in Triton X-100 extraction as well as in guanidine extraction of 22M mPFCs compared to 3M mPFCs (Fig. 1E). Moreover, we assessed the total YTHDF protein levels by homogenizing mPFC tissues directly in 5M guanidine hydrochloride and detected no significant difference for YTHDF2 protein levels between 3M and 22M mPFCs (Fig. 1F). These findings suggest that soluble YTHDF2 transforms into insoluble aggregates during brain aging. Similar transformation from soluble to insoluble form was evident for YTHDF1 protein from 3M to 22M mPFCs (Fig. 1E and 1F). Taken together, aging induces transformation of YTHDF proteins from soluble form into insoluble aggregates.

To mimic age-dependent formation of YTHDF aggregates, we next individually overexpressed YTHDF1-mCherry, YTHDF2-zsGreen, or simultaneously overexpressed YTHDF1-mCherry and YTHDF2-zsGreen in N2a cells. We observed that both YTHDF1 and YTHDF2 formed aggregates in cells expressing YTHDF1-mCherry or YTHDF2-zsGreen (Fig. 1G and 1H). In addition, YTHDF1 and YTHDF2 formed co-aggregates in N2a cells concomitantly expressing both YTHDF1-mCherry and YTHDF2-zsGreen (Fig. 1I). We noted that the YTHDF1–YTHDF2 co-aggregates were more resistant to photobleaching than YTHDF1 or YTHDF2 aggregates, suggesting that YTHDF1–YTHDF2 co-aggregates show poorer fluidity than individual YTHDF1 or YTHDF2 aggregates (Fig. S1E–H). These findings suggest that YTHDF1 and YTHDF2 form insoluble aggregates during brain aging or forced expression of YTHDF1 and YTHDF2.

Phosphorylation is a posttranslational modification for proteins and is known to modulate the stability of protein aggregates (Rezaei-Ghaleh et al., 2016). The YTHDF proteins contain Prion-like LCD domains, that are mostly responsible for the phase-phase separation and aggregate formation of YTHDF proteins (Gao et al., 2019). We found that the LCD regions of YTHDF proteins contain several putative phosphorylation sites (Fig. S2A and S2B). By conducting immunoblotting for the sequential extractions of 22M and 3M mPFCs, we detected a substantial amount of phosphorylated YTHDF2 proteins in 5M guanidine hydrochloride fraction (insoluble) of 22M mPFCs, whereas no such phosphorylated YTHDF2 proteins were detected in the same fraction of 3M mPFCs (Fig. 1J). Of note, the phosphorylation for YTHDF2 protein occurred on its serine (Ser) residues, and no phosphorylation on threonine (Thr) or tyrosine (Tyr) residues was detected (Fig. 1J). Similarly, we detected a large amount of phosphorylated YTHDF1 proteins in 5M guanidine hydrochloride fraction of 22M mPFCs, whereas no phosphorylated YTHDF1 proteins was detected in the same fraction of 3M mPFCs (Fig. 1K). In contrast to YTHDF2, the phosphorylation for YTHDF1 occurred on its Thr residues, and no phosphorylation on its Ser or Tyr residues was detected (Fig. 1K). Moreover, we detected no phosphorylated YTHDF1 and YTHDF2 proteins in Triton X-100 fractions (detergent soluble) (Fig. 1J and 1K). These findings suggest that phosphorylation modification for YTHDF proteins is associated with the formation of insoluble protein aggregates.

To determine whether phosphorylation modification is required for aggregate formation of YTHDF proteins, we next generated a YTHDF1 mutant, wherein all threonine residues on LCD were replaced with alanine, with a flag-tag on its N-terminus (YTHDF1-TM). N2a cells were transfected with YTHDF1 or YTHDF1-TM plasmid, followed by IP with an anti-flag antibody and immunoblotting with an anti-p-Thr antibody. We found that this YTHDF1-TM cannot be phosphorylated and N2a cells expressing YTHDF1-TM showed no formation of YTHDF1 aggregates (Fig. S2C and S2D), suggesting that phosphorylation modification on threonine residues is required for YTHDF1 aggregate formation. We also generated a YTHDF2 mutant with a flag-tag on its N-terminus, wherein all serine residues on LCD were replaced with alanine (YTHDF2-SM). Again, no phosphorylation on YTHDF2-SM was detected by IP with an anti-flag antibody followed by immunoblotting with an anti-p-ser antibody in N2a cells transfected with YTHDF2-SM plasmid (Fig. S2E). Similarly, no formation of YTHDF2 aggregates was detected in N2a cells transfected with YTHDF2-SM plasmid (Fig. S2F), suggesting that phosphorylation modification on serine residues is required for the formation of YTHDF2 aggregates. We noted that N2a cells simultaneously expressing YTHDF1 and YTHDF2-SM displayed no formation of aggregates, whereas N2a cells expressing YTHDF1-TM and YTHDF2 showed a formation of aggregates/co-aggregates (Fig. 1L). These findings suggest that the formation of YTHDF1 and YTHDF2 aggregates requires phosphorylation modification on their LCDs and phosphorylation of YTHDF2 is the driving force for the formation of YTHDF1–YTHDF2 co-aggregates.

**Figure 2.**
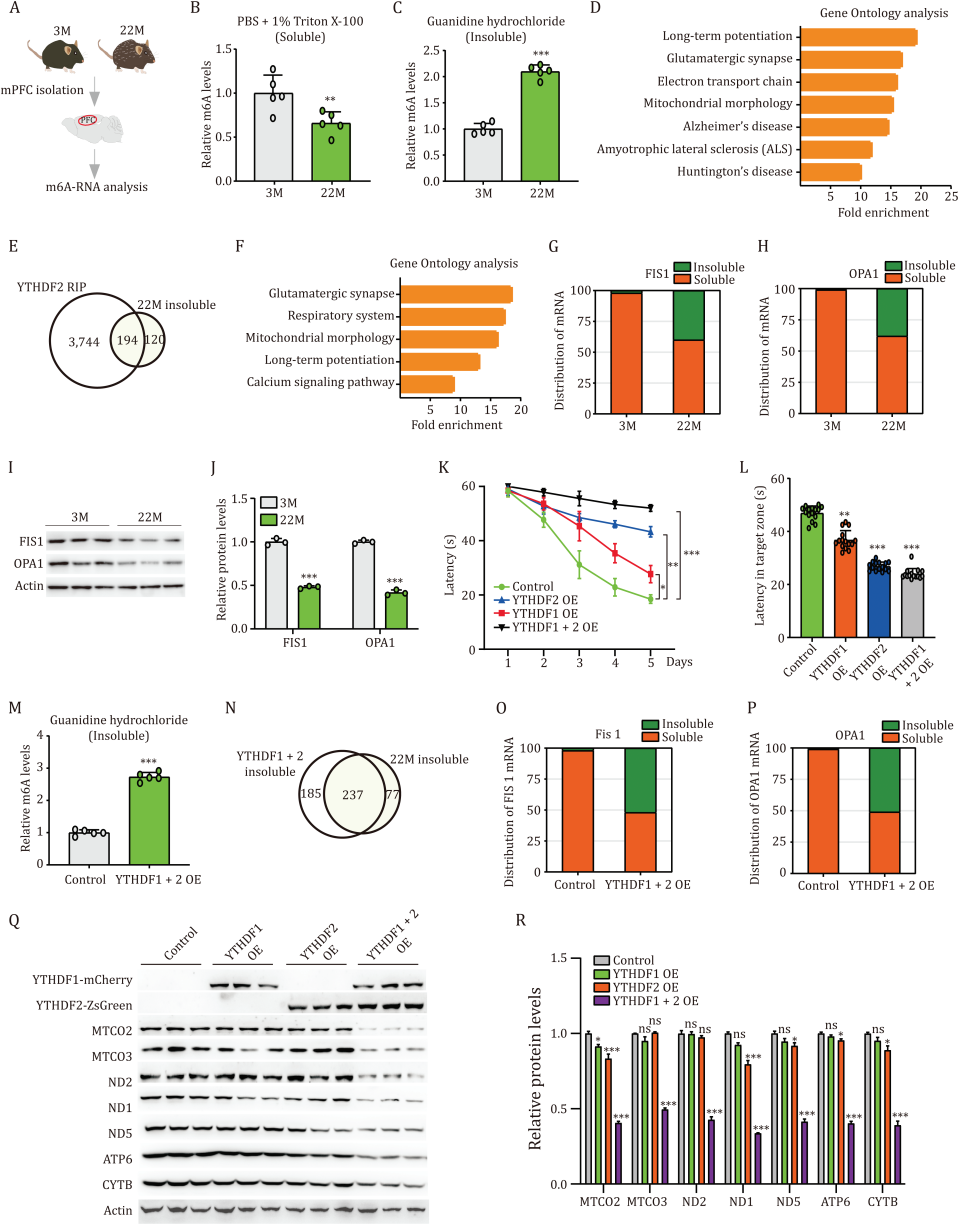
**YTHDF aggregates trap mitochondrial mRNAs and reduce their expression, consequently impair respiratory function and memory.** (A) Schematic of experimental design. (B and C) m6A levels in sequentially extracted 3M and 22M mPFCs by PBS + 1% Triton X-100 (B) and 5M guanidine hydrochloride (C) (*n* = 5). (D) 3M and 22M mPFCs were sequentially extracted by PBS + 1% Triton X-100 and 5M guanidine hydrochloride. RNAs in guanidine hydrochloride fraction were subjected to RNA-seq. Gene Ontology (GO) analysis of significantly up-regulated mRNAs in 22M vs. 3M mPFCs. (E) Venn diagram showing the overlap mRNA numbers between YTHDF2 associated mRNAs in PBS + 1% Triton extracted mPFCs and mRNAs enriched in 5M guanidine hydrochloride extracted 22M mPFCs. (F) GO analysis of overlap mRNAs in (E). (G and H) Distribution of FIS1 (G) and OPA1 (H) mRNA in sequential extracted mPFCs of 3M and 22M mice by PBS + 1% Triton X-100 (soluble) and 5M guanidine hydrochloride (insoluble). (I and J) Protein levels of FIS1 and OPA1 in the mPFCs of 3M and 22M mice, by immunoblotting (I) and densiometric analysis (J) (*n* = 3). (K and L) The Morris water maze task. (K) Spatial learning was evaluated as a function of training day, with respect to escape latency (*n* = 15 mice pergroup). (L) Time spend in the target zone in the probe trial (*n* = 15 mice per group). (M) m6A levels in guanidine hydrochloride extracted mPFCs of control and YTHDF1 + 2 mice (*n* = 5). (N) 22M and YTHDF1 + 2 mice mPFCs were sequentially extracted by PBS + 1% Triton X-100 and 5M guanidine hydrochloride. RNA in guanidine fraction was subjected to RNA-seq. Venn diagram demonstrating the overlap mRNA numbers enriched in the inclusions of 22M and YTHDF1 + 2 mice. (O and P) Distribution of FIS1 (O) and OPA1 (P) mRNA in sequentially extracted mPFCs of control and YTHDF1 + 2 mice, by PBS + 1% Triton X-100 and 5M guanidine hydrochloride. (Q and R) Protein levels of MTCO2, MTCO3, ND2, ND1, ND5, ATP6, and CYTB in the mPFCs of control, YTHDF1, YTHDF2, and YTHDF1 + 2 mice, by immunoblotting (Q) and densiometric analysis (R) (*n* = 3). ns, not significant; ^*^P < 0.05; ^**^P < 0.01; ^***^P < 0.001 by ANOVA or Student’s *t*-test; error bars denote the SEM.

Given that YTHDF proteins are major *N*6-methyladenosine (m6A) readers (Chen et al., 2023), we next investigated the impact of YTHDF aggregates on m6A modified RNAs (Fig. 2A). We found that levels of m6A modified RNAs in Triton X-100 extraction (detergent soluble) of 22M mPFCs was significantly lower than that of 3M mPFCs, whereas levels of m6A modified RNAs in guanidine extraction (insoluble) of 22M mPFCs was significantly higher than that of 3M mPFCs (Fig. 2B and 2C). These findings indicate that YTHDF proteins and their bound RNAs form insoluble inclusions during brain aging.

To categorize the RNA components in the insoluble inclusions, we next sequentially extracted 3M and 22M mPFCs by PBS + 1% Triton X-100 (soluble) and 5M guanidine hydrochloride (insoluble), and guanidine extraction was subjected to RNA-seq analysis. Gene Ontology (GO) analysis on the significantly up-regulated mRNAs (*n* = 314) in 22M vs. 3M mPFCs showed enriched functional annotation related to “Long-term potentiation”, “Glutamatergic synapse”, “Electron transport chain”, and “Mitochondrial morphology” (Fig. 2D), suggesting that at least a subset of mRNAs related to synapse and mitochondrion are trapped in the inclusions.

Given that YTHDF2 is the major driving force for the formation of YTHDF protein aggregates, we next investigated the YTHDF2 associated mRNAs in PBS + 1% Triton X-100 extracted mPFCs (detergent soluble) by conducting IP with an anti-YTHDF2 antibody, followed by RNA sequencing (RIP-seq). An overlap of significantly increased mRNAs in guanidine fraction of 22M vs. 3M mPFCs (*n* = 314) and YTHDF2 bound mRNAs (*n* = 3,938) indicated that 194 out of 314 (62%) significantly increased mRNAs in guanidine fraction of 22M vs. 3M mPFCs were identified as YTHDF2 bound mRNAs (Fig. 2E). GO analysis for these overlap mRNAs showed similarly enriched functional annotation related to synapse and mitochondrion (Fig. 2F), suggesting that at least a subset of synapse and mitochondrion-related mRNAs undergo a YTHDF2-mediated translocation from soluble fraction into fibrotic/insoluble inclusions during brain aging.

Mitochondrial morphology displays defective changes during brain aging (Faitg et al., 2021). By measuring the mRNA levels, we confirmed the transfer of multiple mitochondrial mRNAs, including FIS1 and OPA1 mRNA, from Triton X-100 fraction to guanidine fraction during brain aging (Fig. 2G and 2H). Of note, these mRNA-encoded proteins are known to be related to the establishment of mitochondrial morphology (Pellegrini and Scorrano, 2007). By conducting immunoblotting, we found these mRNA-encoded proteins were significantly reduced in 22M mPFCs compared to 3M mPFCs (Fig. 2I and 2J). These findings suggest that YTHDF proteins and YTHDF2-bound mRNAs form insoluble inclusions, which traps mitochondrial mRNAs and suppresses their expression during brain aging.

To investigate the impact of YTHDF aggregates on brain function, we next conducted overexpression of YTHDF1, YTHDF2, or YTHDF1 and YTHDF2 (YTHDF1 + 2) in the mPFCs of 3M mice. By conducting immunostaining, we verified the existence of YTHDF aggregates in the mPFCs of mice with individual overexpression of YTHDF1 or YTHDF2, or concomitantly overexpression of YTHDF1 and YTHDF2 (Fig. S3A). Behavioral analyses on mice with overexpression of YTHDF1 or YTHDF2 showed impairment in spatial, contextual, and fear memory compared to control mice, wherein mice with YTHDF2 overexpression displayed more severe memory deficits than mice with YTHDF1 overexpression (Figs. 2K, 2L, and S3B–E). Of note, YTHDF1 + 2 mice performed worse than YTHDF2 or YTHDF1 mice in these behavioral tasks (Figs. 2K, 2L, and S3B–E). Specifically, in the Morris water maze task, YTHDF1 + 2 mice exhibited significantly increased time to locate the platform and crossed the platform area less frequently than YTHDF1 mice, YTHDF2 mice, and control mice (Figs. 2K, 2L, and S3B). In novel object recognition task, YTHDF1 + 2 mice spent significantly less time in exploring novel objects than control mice (Fig. S3C). In eight-arm radial maze, YTHDF1 + 2 mice exhibited significantly increased working memory errors compared to control mice (Fig. S3D). In fear conditioning task, YTHDF1 + 2 mice showed significantly less freezing time than control mice (Fig. S3E). These findings suggest that forced expression of YTHDF1 and YTHDF2 induces formation of protein aggregates, that mimic brain aging and impair memory in mice.

To categorize the RNA components of the inclusions formed in the mPFCs of YTHDF1 + 2 mice, we sequentially extracted mPFCs of control and YTHDF1 + 2 mice by PBS + 1% Triton X-100 and 5M guanidine hydrochloride. We found that levels of m6A modified RNAs were significantly increased in guanidine extracted mPFCs of YTHDF1 + 2 mice, compared to that of control mice (Fig. 2M). We also identified a number of mRNAs that were enriched in the inclusions both from YTHDF1 + 2 mPFCs and 22M mPFCs (Fig. 2N), suggesting that brains with forced expression of YTHDF1 + 2 display the translocation of similar set of mitochondrial mRNAs as aging brains. By conducting qPCR analysis, we confirmed the transfer of multiple mitochondrial mRNAs in the mPFCs of YTHDF1 + 2 mice, including FIS1 and OPA1 mRNA, from the detergent soluble fraction to the insoluble fraction, where mRNAs are incapable for translation (Fig. 2O and 2P). Of note, these mRNAs were retained primarily in the soluble fraction in age-matched control mice (Fig. 2O and 2P). Consequently, we detected a significant reduction in FIS1 and OPA1 protein levels in the mPFCs of YTHDF1 + 2 mice compared to that of control mice (Fig. S3F and S3G). These findings support that YTHDF aggregates decrease mitochondrial gene expression by trapping their mRNAs in the insoluble inclusions and reducing their expression.

We next investigated the impact of YTHDF aggregates on mitochondria. Immunostaining indicated that TOM20, a mitochondrial marker protein, was significantly reduced in the mPFCs of YTHDF1 or YTHDF2 mice (Fig. S4A). A greater extent of reduction was observed in the mPFCs of YTHDF1 + 2 mice (Fig. S4A). Moreover, TOM20 was found significantly reduced in N2a cells with YTHDF1 or YTHDF2 overexpression, and a further reduction was observed in N2a cells with overexpression of YTHDF1 + 2, compared to control N2a cells (Fig. S4B). Reduction in TOM20 was also evident in N2a cells with YTHDF1 + 2 overexpression by immunostaining (Fig. S4C). These findings indicate that YTHDF aggregates decrease mitochondrial biogenesis.

In addition, major mitochondrial respiratory chain complex components also showed a significant reduction in the mPFCs of YTHDF1 + 2 mice, including MTCO2, MTCO3, ND1, ND2, ND5, ATP6, and CYTB compared to control mice (Fig. 2Q). We noted that their mRNA levels were also significantly reduced in the mPFCs of YTHDF1 + 2 mice, compared to that of control mice (Fig. S4D). Similar reduction in protein and mRNA levels of these genes was also evident in N2a cells with YTHDF1 + 2 overexpression (Fig. S4E–G). Consequently, ATP production was significantly reduced in the mPFCs of YTHDF1 + 2 mice as well as in N2a cells with YTHDF1 + 2 overexpression (Fig. S4H and S4I). These findings suggest that YTHDF aggregates decrease mitochondrial respiratory function through reducing mitochondrial biogenesis.

Mitochondria continuously join by the process of fusion and divide by the process of fission (Westermann, 2010). We found that levels of mitofusion 1 (MFN1) and mitofusion 2 (MFN2), both mediate mitochondrial fusion processes, were significantly decreased in the mPFCs of YTHDF1 + 2 mice (Fig. S5A and S5B). Moreover, we observed that levels of dynamin-related protein 1 (DRP1) and its active form p-DRP1, mitochondrial fission mediators, were significantly reduced in the mPFCs of YTHDF1 + 2 mice (Fig. S5A and S5B). Reductions in DRP1 targeting proteins, such as mitochondrial fission protein 1 (FIS1) and mitochondrial fission factor (MFF), were also observed in the mPFCs of YTHDF1 + 2 mice (Fig. S5A and S5B). Similar changes in mitochondrial dynamics were also evident in N2a cells with YTHDF1 + 2 overexpression (Fig. S5C and S5D). We noted that their mRNA levels did not differ between control and YTHDF1 + 2 mPFCs or between control and YTHDF1 + 2 expressing N2a cells (Fig. S5E and S5F). These findings suggest that YTHDF aggregates impair mitochondrial dynamics.

In conclusion, YTHDF proteins form insoluble aggregates during brain aging, these aggregates trap a set of mitochondrial mRNAs and reduce their expressions, consequently impair mitochondrial biogenesis, dynamics, respiratory function, and memory in mice. Thus, through illustrating a role for YTHDF aggregates in disrupting mitochondrial function during brain aging, our study defines YTHDF aggregates as a potential hallmark for brain aging. YTHDF aggregates may serve as a therapeutic target for age-related cognitive decline.

## Supplementary Material

pwad041_suppl_Supplementary_Materials

## Footnotes

This research was supported by the National Natural Science Foundation of China (Grant Nos. 82125009, U19A2034, 31871082, 91849101, 32121002, 82071185, 32100794 and 92149303), the National Key R&D Program of China (2020YFA0509300, 2021YFA0804900), the Strategic Priority Research Program of the Chinese Academy of Sciences (XDB39000000), CAS Project for Young Scientists in Basic Research (YSBR-013), Collaborative Innovation Program of Hefei Science Center, CAS (2022HSC-CIP003), the Fundamental Research Funds for the Central Universities (YD2070002011, WK2070000168), and Institute of Health and Medicine, Hefei Comprehensive National Science Center (QYZD20220003).

The authors declare no competing interests.

All animal procedures were approved by and performed in accordance with the guidelines of the Institutional Animal Care and Use Committee of University of Science and Technology of China (USTCACUC26100122062).

RNA-seq and RIP-seq data are deposited to GEO (GSE233453).

J.Z., D.L., Q.L., and Z.X. conceived the project, designed the experiments, and wrote the manuscript. J.Z., D.L., and K.H. conducted the experiments and data analysis.
