# Supplementary material for: Aging-induced YTHDF aggregates impair mitochondrial function by trapping mitochondrial RNAs and suppressing their expression in the brain
Source: Protein Cell. 2023 Jul 4;15(2):149–55. doi: 10.1093/procel/pwad041 (PMC10833457; doi:10.1093/procel/pwad041)
Supplement: pwad041_suppl_Supplementary_Materials [file pwad041_suppl_supplementary_materials.pdf]

**Supplemental information for**

# Aging-induced YTHDF aggregates impair mitochondrial function by trapping mitochondrial RNAs and suppressing their expression in the brain

**Key Resource Table**

**Supplementary Table 1**

**Materials and Methods**

**References**

**Figure legends S1 – S5**

**Figure S1**

**Figure S2**

**Figure S3**

**Figure S4**

**Figure S5**

## Key Resource Table (REAGENT or RESOURCE)

| REAGENT or RESOURCE | SOURCE | IDENTIFIER |
|---|---|---|
| **Antibodies** | | |
| Rabbit polyclonal anti-Actin | Proteintech | Cat# 66009-1-Ig; **RRID**: AB_2919667; **WB:** 1:5,000 |
| Mouse monoclonal anti-Flag | Elabscience | Cat# E-AB-20006; **WB:** 1:5,000; **IP:** 2.5μg |
| Mouse monoclonal anti-p-Ser | Santa Cruz | Cat# sc-81515; **RRID**: AB_1128625; **WB:** 1:500 |
| Mouse monoclonal anti-p-Thr | Santa Cruz | Cat# sc-5267; **RRID**: AB_628121; **WB:** 1:500 |
| Mouse monoclonal anti-p-Tyr | Santa Cruz | Cat# sc-7020; **RRID**: AB_628123; **WB:** 1:500 |
| Rabbit polyclonal anti-TOM20 | Proteintech | Cat# 11802-1-AP; **RRID**: AB_2919606; **WB:** 1:5,000 |
| Rabbit polyclonal anti-ND1 | Proteintech | Cat# 19703-1-AP; **RRID**: AB_10637853; **WB:** 1:1,000 |
| Rabbit polyclonal anti-ND2 | Proteintech | Cat# 19704-1-AP; **RRID**: AB_10638920; **WB:** 1:1,000 |
| Rabbit polyclonal anti-ND3 | Enogene | Cat# E917969; **WB:** 1:500 |
| Rabbit polyclonal anti-ND5 | Proteintech | Cat# 55410-1-AP; **RRID**: AB_2881324; **WB:** 1:1,000 |
| Rabbit polyclonal anti-CYTB | Proteintech | Cat# 55090-1-AP; **RRID**: AB_2881266; **WB:** 1:1,000 |
| Rabbit polyclonal anti-MTCO1 | Abclonal | Cat# A17889; **RRID**: AB_2861744; **WB:** 1:1,000 |
| Rabbit polyclonal anti-MTCO2 | Proteintech | Cat# 55070-1-AP; **RRID**: AB_10859832; **WB:** 1:1,000 |
| Rabbit polyclonal anti-MTCO3 | Proteintech | Cat# 55082-1-AP; **RRID**: AB_2881265; **WB:** 1:1,000 |
| Rabbit polyclonal anti-ATP6 | Proteintech | Cat# 55313-1-AP; **RRID**: AB_2881305; **WB:** 1:500 |
| Rabbit polyclonal anti-OPA1 | Proteintech | Cat# 27733-1-AP; **RRID**: AB_2810292; **WB:** 1:3,000 |
| Rabbit polyclonal anti-FIS1 | Proteintech | Cat# 10956-1-AP; **RRID**: AB_2919755; **WB:** 1:3,000 |
| Rabbit polyclonal anti-MFN1 | Proteintech | Cat# 13798-1-AP; **RRID**: AB_2266318; **WB:** 1:3,000 |
| Rabbit polyclonal anti-MFN2 | Proteintech | Cat# 12186-1-AP; **RRID**: AB_2266320; **WB:** 1:3,000 |
| Rabbit polyclonal anti-MFF | Proteintech | Cat# 17090-1-AP; **RRID**: AB_2142463; **WB:** 1:5,000 |
| Rabbit polyclonal anti-DRP1 | Proteintech | Cat# 12957-1-AP; **RRID**: AB_2934878; **WB:** 1:5,000 |
| anti-DRP1 (phospho Ser637) antibody | GeneTex | Cat# GTX01567; **WB:** 1:2,000 |
| Goat anti-mouse Alexa Fluor 488 | Invitrogen | A11001 |
| Goat anti-mouse Alexa Fluor 594 | Invitrogen | A11032 |
| Goat anti-rabbit Alexa Fluor 488 | Invitrogen | A11034 |
| Goat anti-rabbit Alexa Fluor 594 | Invitrogen | A11037 |
| **Plasmids** | | |
| pLVX-zsGreen | Clontech | 632565 |
| pLVX-mCherry | Clontech | 632561 |
| pcDNA3.1 | Invitrogen | V79020 |
| **Critical Commercial Assays** | | |
| ATP determination kit | Beyotime | Cat# S0026 |

| | | |
|---|---|---|
| EpiQuik m6A RNA Methylation Quantification Kit | Epigentek | Cat #P-9005-96 |
| **Software and Algorithms** | | |
| Image J | NIH | Version 1.53c |
| GraphPad Prism | GraphPad | Version 8 |
| SuperFcs XR | Shanghai XinRuan Information Technology | http://www.softmaze.com |
| Ethovision XT 11 | Noldus | https://www.noldus.com/ethovision-xt |

## Supplementary Table 1 (Oligo sequences)

| **qPCR detection primers** | | |
|---|---|---|
| **FIS1** | F | TGTCCAAGAGCACGCAATTTG |
| | R | CCTCGCACATACTTTAGAGCCTT |
| **OPA1** | F | CGACTTTGCCGAGGATAGCTT |
| | R | CGTTGTGAACACACTGCTCTTG |
| **MTCO2** | F | CTAATTAGCTCCTTAGTCCTC |
| | R | TTCGTAGCTTCAGTATCATTG |
| **MTCO3** | F | ATTCTATTCATCGTCTCGGAA |
| | R | AAGGCTATGATGAGCTCATGT |
| **ND1** | F | TTACCAGAACTCTACTCAACT |
| | R | ATCGTAACGGAAGCGTGGATA |
| **ND2** | F | TCAATAATTATCCTCCTGGCC |
| | R | ATGATAGTAGAGTTGAGTAGC |
| **MFN1** | F | ATGGCAGAAACGGTATCTCCA |
| | R | CTCGGATGCTATTCGATCAAGTT |
| **DRP1** | F | CAGGAATTGTTACGGTTCCCTAA |
| | R | CCTGAATTAACTTGTCCCGTGA |
| **MFF** | F | AGCTGCCGCCACTTCTAATC |
| | R | TGCATCTACCACAGTCATGTCA |
| **ND5** | F | AACCACACCTAGCATTCCTAC |
| | R | CAGGCGTTGGTGTTGCAGGTA |
| **MFN2** | F | GTGGGCTGGAGACTCATCG |
| | R | CTCACTGGCGTATTCCGCAA |
| **ATP6** | F | TAATCAACAACCGTCTCCATTC |
| | R | GTGTCGGAAGCCTGTAATTAC |
| **CYTB** | F | GCAACGAAGCCTAATATTCC |
| | R | TGAGATTGGTATAAGAATTAA |
| **Actin** | F | TGCTGTCCCTGTATGCCTCTG |
| | R | TGATGTCACGCACGATTTCC |

| Cloning primers | | |
|---|---|---|
| **YTHDF1** | F | GCCACCATGTCGGCCACCAGCGT |
| | R | TTATTGTTTGTTTCGATTCTGTCTTTCC |
| **YTHDF2** | F | GCCACCATGTCGGCCAGCAGCCTC |
| | R | CTATTTCCCACGACCTTGACGTT |
| **YTHDF1-M1** | F | ATGTCGGCCACCAGCGTG |
| | R | AGGGCTCTGATACTGTGGCTG |
| **YTHDF1-M2** | F | CAGCCACAGTATCAGAGCCCT |
| | R | TTATTGTTTGTTTCGATTCTGTCTTTCC |
| **YTHDF1-M3** | F | CAGCCACAGTATCAGAGCCCT |
| | R | AAACCAGGTCGGTGTCATAGTCTCGGGA |
| **YTHDF1-M4** | F | ATGTCGGCCACCAGCGTG |
| | R | TCCCACCATTGCCAGAAAGGACA |
| **YTHDF2-M1** | F | ATGTCGGCCAGCAGCCTCTTGGA |
| | R | TGCCTGAGCCACTGGTGGGC |
| **YTHDF2-M2** | F | AGCCCACCAGTGGCTCAGGCA |
| | R | CTATTTCCCACGACCTTGACGTT |
| **YTHDF2-M3** | F | GGTCCATCACTAGTAACATTGTGG |
| | R | CTGCCTGAGCCACTGGTGGGCT |
| **YTHDF2-M4** | F | ATGTCGGCCAGCAGCCTCTTGGA |
| | R | TTTGAACCAAAGCCTGTGAGGG |
| **YTHDF1-TM** | Part 1-S-F | GCCACCATGTCGGCCACCAGCGT |
| | Part 1-S-R | CAGAAAGGACACCGGCCAGTGCCACACTG TTGACAACTGAACCCACCG |
| | Part 2-S-F | GACGGTGGGTTCAGTTGTCAACAGTGTGGC ACTGGCCGGTG |
| | Part 2-S-R | CCAGGACAGGGTGGGATTCTACACTCGGG GCAGAGGCAGGT |
| | Part 3-S-F | AAATGCCCAACCTGCCTCTGCCCCGAGTGT AGAATCCCACCCTG |
| | Part 3-S-R | TTATTGTTTGTTTCGATTCTGTCTTTCC |
| **YTHDF2- SM** | Part 1-S-F | GCCACCATGTCGGCCAGCAGCCTC |
| | Part 1-S-R | CTACAACTTTTGGAACGGCGGCTGCAACTT CTGTGCTACCTAGTTTCAGT |
| | Part 2-S-F | ACTGAAACTAGGTAGCACAGAAGTTGCAG CCGCCGTTCCAAAAGT |
| | Part 2-S-R | ACACCGGGTGAGGCTCTGAAGGAGTGGCT CCGGCACCCGCC |
| | Part 3-S-F | ACAGGCCCAGGCGGGTGCCGGAGCCACTC CTTCAGAGCCTCACCC |
| | Part 3-S-R | CTATTTCCCACGACCTTGACGTT |

# Materials and Methods

## Experimental Model and Subject Details

### Animals

C57BL/6J mice were purchased from Charles River Laboratories. All experimental protocols were approved by the Animal Studies Committee at University of Science and Technology, Hefei, China.

### Cell lines

Mouse N2a cells (ATCC, CCL-131) and 293T cells (ATCC, CRL-11268) were cultured under standard conditions in DMEM (Gibco) supplemented with 10% FBS (Gibco), 1% penicillin/streptomycin (Invitrogen) and incubated in 5% humidified $CO_2$ incubator at 37°C.

## Method Details

### Plasmid Construction

YTHDF1 and YTHDF2 coding sequences were PCR amplified from mouse mPFC cDNA, and then subcloned into BamHI/EcoRI restriction sites of pcDNA3.1 backbone plasmid (Invitrogen) and pLVX-mcherry or pLVX-ZsGreen backbone plasmid (Clontech). Truncation fragments of YTHDF1 (YTHDF1-M1, YTHDF1-M2, YTHDF1-M3, YTHDF1-M4) and YTHDF2 (YTHDF2-M1, YTHDF2-M2, YTHDF2-M3, YTHDF2-M4) with HA and Flag tag on the N-terminus were subcloned into pcDNA3.1 backbone plasmid. YTHDF1-TM mutant was generated by replacing all threonine residues on the low complexity domain (LCD) with alanine, with a flag-tag on the N-terminus. YTHDF2-SM mutant was generated by replacing all serine residues on the low complexity domain (LCD) with alanine, with a flag-tag on the N-terminus. Cloning primers are listed in Supplementary Table 1.

### Immunofluorescence Staining

N2a cells grown on cover slips were washed with PBS for 5 min at RT, fixed by 4% PFA in PBS for 10 min, and permeabilized with 0.4% Triton X-100 in PBS for 10 min. Mice were perfused transcardially with PBS, brains were immersed in 4% paraformaldehyde (PFA) and then cryo-preserved in 30% sucrose for 24 h at 4°C. Tissues were then embedded in OCT compound and sectioned with microtome at 40 mm thickness (Leica). Glass slide-mounted sections were washed with PBS, permeabilized with PBS containing 0.25% Triton X-100. Cells and brain sections were subjected to blocking with PBS containing 1% BSA and 0.5% Triton X-100 for 30 min at 37°C, and then washed with PBS. This is followed by incubation with primary antibodies at RT for 3 h, and then with Alexa 488 and or Alexa 594 labeled secondary antibodies (Invitrogen). Images were acquired using a Leica TCS SPE confocal microscope equipped with DFC 365 FX Digital Camera.

### Immunoblotting and Densitometric analysis

Cells and mPFC tissues were lysed on ice in lysis buffer (PBS plus 1% Triton X-100 and 1% proteinase inhibitor (MCE), then sonicated for 10 min. Cell and mPFC lysates were centrifuged at 14,000 rpm for 15 min and protein concentration in the supernatant was determined using a BCA Protein Assay kit (Pierce). Equal amount of protein was separated by SDS-PAGE electrophoresis,

and then transferred to nitrocellulose membrane (Poll). Membranes were blocked with 5% non-fat milk in tris buffer saline (TBS) containing 0.1% Tween 20 (TBST) for 1 h, followed by antibody incubation at 4°C overnight. The immunoreactive bands were visualized by enhanced chemiluminescence (Pierce) using ChemiScope (CLiNX). For densitometric analyses, immunoreactive bands were quantified by Image J software.

## Co-immunoprecipitation (Co-IP)

Cells were pelleted by centrifugation at 1,500 rpm for 5 min at 4°C, and then resuspended in MCLB buffer (50 mM Tris pH 8.0, 5 mM EDTA, 0.5% NP-40, 100 mM NaCl) for 20 min at 4°C with end-over-end rotation. The cell lysates were sonicated for 5 min (2s on, 1s off) and centrifuged at 14,000 rpm for 10 min at 4°C. Supernatant was saved and protein concentration was determined by BCA kit (Pierce) according to manufacturer's instructions. The primary antibody was incubated with protein A/G magnetic beads (MCE) at room temperature for 2 h, and then incubated with cell lysates at 4°C overnight. The protein A/G magnetic beads were washed with PBS and eluted by protein loading buffer (10% SDS, 500 mM DTT, 50% glycerol, 500 mM Tris-HCL, 0.5% bromophenol blue dye). The eluted proteins were subjected to SDS-PAGE gel electrophoresis and immunoblotting.

## RNA extraction, Reverse transcription, and Quantitative PCR

Total RNA was extracted from cell or tissue using Trizol (Invitrogen), according to manufacturer's protocol. RNA was then reverse transcribed using HiScript® II Reverse Transcriptase (Vazyme) in the presence of an anchor RT primer. For mRNA detection, quantitative PCR was conducted with AceQTM qPCR SYBR Green Master Mix (Vazyme) on LightCycler 96 system (Roche) according to standard procedures. The measured value for each sample was averaged and compared using CT method. qPCR detection primers were listed in Supplementary Table 1.

## Generation of Adeno-associated virus and mPFC Injection

Adeno-associated virus (AAV) was produced according to previously published[1]. Briefly, target sequences in AAV vector, pHelper, and AAV serotype 9 packaging plasmid were introduced into 293T cells in a ratio of 2:1:1 by transfection with polyethylenimine (PEI, Sigma). 24 h after transfection, medium was replaced with DMEM plus 2% FBS. 72 h after transfection, both cells and medium were collected for AAV purification. AAV particles were released from cells with freeze/thaw cycles, followed by incubation with 50 U/ml benzonase nuclease (MKbio) and 10 U/ml RNase I (Vazyme) at 37°C for 30 min. Incubation continues for another 30 min upon adding of 0.5% sodium deoxycholate (Sigma), cell debris were removed by centrifugation at 2,500 g for 30 min. 40% PEG8000 and 2.5 M NaCl were added to precipitate the virus. The pellet was re-suspended in PBS, followed by chloroform and $(NH_4)_2SO_4$ extraction to remove contaminated proteins. Viral titer was determined by qPCR-based approach. Male wild-type C57BL/6J mice at 8 weeks of age were stereotaxically injected with AAV virus into the mPFC with an air pressure injector system (KDS). The coordinates used for stereotaxic injections were AP +1.9, ML 0.3, DV -1.7 and AP +1.9, ML -0.3, DV -1.7. Behavioral tests and other assays were conducted 4 weeks after the injection.

## Behavioral assays

## The Morris Water Maze

The Morris water maze was performed according to a previously published[2]. Briefly, mice were

trained in the water tank to navigate a direct path to the hidden escape platform, when mice were released from semi-random locations around the perimeter of the tank. During the training phase, each individual mouse received consecutive trials for continuous 5 days, followed by a probe trial. On probe trial day, the platform was removed and mice were allowed to swim for 90 seconds starting from the quadrant opposite to the quadrant where the platform had been located. Behavioral parameters were recorded by a video camera set on top of the water tank and data were analyzed using Ethovision XT 11 software (Noldus).

## Contextual Fear Conditioning

The contextual fear conditioning was conducted according to previously published[3]. Briefly, on day 1, mice were allowed to explore the chamber for 3 min and then exposed to 20 sec tone (85 dB, 2700 Hz) (conditioned stimulus), after a trace period of 20 sec a mild foot shock (2 sec, 0.5 mA) (unconditioned stimulus) was administered to these mice. Five conditioning trials (pairing) were performed with a 200 sec inter-trial interval. On day 2, trace memory was evaluated. A 2 min baseline period followed by three 20 sec tones with 220 sec intervals was presented to mice, and freezing behavior was recorded accordingly. On day 3, contextual memory was assessed. The context should be identical to that of day 1. Freezing behavior was recorded for 8 min. Behavioral parameters were recorded by a video camera and data were analyzed using Ethovision XT 11 software (Noldus).

## Novel Object Recognition (NOR)

The object-context discrimination task was performed as described[4], with modifications. Briefly, mice were placed in an open chamber with a specific floor pattern and two identical objects, followed by 10 min exploration and an inter-trail interval (trial one). Mice then were placed in a second chamber with different floor pattern and the objects unique from the objects in the first trial (trial two). Mice were finally tested for 10 min in a chamber consisting of a floor pattern from either trial one or trial two, one object from trial one, and another object from trial two. The time mice spent in exploring the object in novel context was compared to the same object in the old context. Behavioral parameters were recorded by a video camera and data were analyzed using Ethovision XT 11 software (Noldus).

## Radial Arm Maze

The eight-arm radial maze task was performed as described[5]. Briefly, day 1- mice were subjected to habituation at the apparatus for 10 min without food at the end of the arms. Day 2 - mice were subjected to food deprivation until when the animals had arrived at the 80%–85% of their initial weight. Day 3 - Training: food was placed in four non-adjacent arms at the end of each arm, and mice were released in the center of the arena. Mice entering at least two arms containing food pellets was considered successful learning. Day 4 to 13 - Test: The food pellets were placed only in one of the eight-arm apparatus, and each mouse was released in the center of the arena. Frequencies to enter the arm containing food pellets and arms containing no food pellet were recorded and plotted. The maze was cleaned up with water and 70% ethanol before the next mouse was placed in the apparatus. Behavioral parameters were recorded by a video camera and data were analyzed using Ethovision XT 11 software (Noldus).

## Measurement of ATP Concentration

ATP concentration was measured in the mPFC lysates or cells using a luciferin/luciferase bioluminescence assay kit (ATP determination kit # C0068M, Beyotime Biotechnology), according to the manufacturer's instructions. The amount of ATP in each sample was calculated from standard curves and normalized to the total protein concentration.

## m6A RNA Methylation Assay

m6A RNA methylation was measured in the mPFC lysates using EpiQuik m6A RNA Methylation Quantification Kit (#P-9005-96, Epigentek), according to the manufacturer's instructions. Briefly, the total RNA was added to strip wells with RNA high binding solution, followed by addition of an anti-m6A antibody to each well to capture m6A. After incubation at RT for 60 min, detection antibody was added to each well to detect m6A signal. Signals were quantified by a spectrophotometer (Molecular Devices).

## Statistical Analysis

All quantified data represent an average of at least triplicate samples. Statistical significance was determined by Student's *t*-test or two-way ANOVA in GraphPad Prism 8.0. $P < 0.05$ was considered significant (indicated by an asterisk in the figures), $P < 0.01$ (indicated by two asterisks in the figures), $P < 0.001$ (indicated by three asterisks in the figures), ns not significant.

## References


1. Guo, P., El-Gohary, Y., Prasadan, K., Shiota, C., Xiao, X., Wiersch, J., Paredes, J., Tulachan, S., and Gittes, G.K. Rapid and simplified purification of recombinant adeno-associated virus. J Virol Methods. 183, 139-146 (2012).
2. Vorhees, C. V. & Williams, M. T. Morris water maze: procedures for assessing spatial and related forms of learning and memory. Nat Protoc. 1, 848-58 (2006).
3. Lugo, J. N., Smith, G. D. & Holley, A. J. Trace fear conditioning in mice. J Vis Exp. (2014).
4. Jain, S. et al. Arf4 determines dentate gyrus-mediated pattern separation by regulating dendritic spine development. PLoS One. 7, e46340 (2012).
5. Sessa, A., Fagnocchi, L., Mastrototaro, G., Massimino, L., Zaghi, M., Indrigo, M., Cattaneo, S., Martini, D., Gabellini, C., Pucci, C., Fasciani, A., Belli, R., Taverna, S., Andreazzoli, M., Zippo, A., & Broccoli, V. SETD5 Regulates Chromatin Methylation State and Preserves Global Transcriptional Fidelity during Brain Development and Neuronal Wiring. Neuron. 104(2), 271–289 (2019).

**Figure S1. YTHDF1 and YTHDF2 proteins interact through their Pro/Gln domains and YTHDF1-YTHDF2 co-aggregates show poorer fluidity than individual aggregates.** (A-B) Schematics of full-length YTHDF1 (A) or YTHDF2 (B) protein and their YTH and Pro/Gln rich domains, truncation fragments M1 (missing YTH domain on the C-terminus), M2 (containing only YTH domain), M3 (containing only Pro/Gln rich domain), and M4 (missing both YTH and Pro/Gln domains). All truncation fragments were generated with HA and Flag tags on the N-terminus. (C-D) YTHDF1 (C) or YTHDF2 (D) truncation fragments M1, M2, M3, and M4 were transfected into N2a cells, followed by immunoprecipitation with an anti-flag antibody and detection with anti-YTHDF2 (C) or anti-YTHDF1 (D) antibodies. (E) Representative images of fluorescence recovery after photobleaching (FRAP) measurement of mCherry and ZsGreen fluorescence *in vivo* (n=3). Yellow squares indicate the same area before (pre-bleach), at 10, 20, and 60 seconds (s) after photobleaching. Scale bar: 10 μm. (F-H) FRAP curves of mCherry (F), ZsGreen (G), and mCherry+ZsGreen (H) fluorescence over a 120 s period (n=3).

**Figure S2. Formation of YTHDF1 and YTHDF2 aggregates requires phosphorylation modification on their low complexity domains.** (A-B) Schematic of YTHDF1 (A) or YTHDF2 (B) protein and their putative phosphorylation sites (as indicated by red bars). (C-D) N2a cells were transfected with YTHDF1 or YTHDF1-TM plasmid for expression. (C) Levels of p-Thr-YTHDF1, detected by IP with an anti-flag antibody and immunoblotting with an anti-p-Thr antibody. (D) Representative fluorescence images of YTHDF1 (green), YTHDF1-TM (green), and DAPI (blue). Scale bar: 10 μm. (E-F) N2a cells were transfected with YTHDF2 or YTHDF2-SM plasmid for expression. (E) Levels of p-Ser-YTHDF2, detected by IP with an anti-flag antibody and immunoblotting with an anti-p-Ser antibody. (F) Representative fluorescence images of YTHDF2 (green), YTHDF2-SM (green), and DAPI (blue). Scale bar: 10 μm.

**Figure S3. YTHDF1 and YTHDF2 positive aggregates impair memory in mice.** (A-F) YTHDF1-mCherry, YTHDF2-zsGreen, or YTHDF1-mCherry+YTHDF2-zsGreen plasmids were delivered to the mPFCs of mice for expression by AAV-mediated delivery technology. (A) Representative fluorescence images of YTHDF1 (red), YTHDF2 (green), and DAPI (blue) in the mPFCs of control, YTHDF1, YTHDF2, and YTHDF1+2 mice. Scale bar: 10 μm. (B) In the Morris water maze task, frequency of target quadrant crossing in the probe trial (n=15 mice per group). (C) Pattern separation memory assessed in a novel-object-recognition task. Plot of object exploration time (n=15 per group). (D) Eight-arm radial maze task. The mean error was plotted (n=15 per group). (E) Fear-conditioning task. The percentage of freezing time during the tone test, assessed following trace fear conditioning (n=15 per group). (F-G) Protein levels of FIS1 and OPA1 in the mPFCs of control and YTHDF1+2 mice, by immunoblotting (F) and densiometric analysis (G) (n=3). ns not significant; *P<0.05; **P<0.01; ***P<0.001 by ANOVA or Student's *t*-test; error bars denote the SEM.

**Figure S4. YTHDF1 and YTHDF2 positive aggregates impair mitochondrial biogenesis and respiratory function in mice.** (A) Protein levels of TOM20 in the mPFCs of control, YTHDF1,

YTHDF2, and YTHDF1+2 mice, by immunoblotting and densiometric analysis (n=3). (B) N2a cells were transfected with YTHDF1, YTHDF2, or YTHDF1+2 plasmids for expression. Protein levels of TOM20, by immunoblotting and densiometric analysis (n=3). (C) N2a cells were transfected with control or zsGreen-YTHDF1+CFP-YTHDF2 plasmids for expression. Representative immunofluorescence images of TOM20 (red), YTHDF1 (zsGreen), YTHDF2 (CFP), and DAPI (blue) in control and YTHDF1+2 N2a cells. Scale bar: 20 μm. (D) mRNA levels of MTCO2, MTCO3, ND2, ND1, ND5, ATP6, and CYTB in the mPFCs of control, YTHDF1, YTHDF2, and YTHDF1+2 mice, by qPCR analysis (n=3). (E-G) N2a cells were transfected with YTHDF1, YTHDF2, or YTHDF1+2 plasmids for expression. (E-F) Protein levels of MTCO2, MTCO3, ND2, ND1, ND5, ATP6 and CYTB, by immunoblotting (E) and densiometric analysis (F) (n=3). (G) mRNA levels of MTCO2, MTCO3, ND2, ND1, ND5, ATP6, and CYTB, by qPCR analysis (n=3). (H) ATP levels in the mPFCs of control, YTHDF1, YTHDF2, and YTHDF1+2 mice (n=3). (I) ATP levels in N2a cells transfected with YTHDF1, YTHDF2, or YTHDF1+2 plasmids (n=3). ns not significant; *P<0.05; **P<0.01; ***P<0.001 by ANOVA or Student's *t*-test; error bars denote the SEM.

**Figure S5. YTHDF1 and YTHDF2 positive aggregates impair mitochondrial dynamics.** (A-B) Protein levels of MFN1, MFN2, DRP1, p-DRP1, FIS1, and MFF in the mPFCs of control, YTHDF1, YTHDF2, and YTHDF1+2 mice, by immunoblotting (A) and densiometric analysis (B) (n=3). (C-D) Protein levels of MFN1, MFN2, p-DRP1, DRP1, FIS1, and MFF in N2a cells with overexpression of YTHDF1, YTHDF2, or YTHDF1+2, by immunoblotting (C) and densiometric analysis (D) (n=3). (E) mRNA levels of MFN, MFN2, DRP1, FIS1, OPA1, and MFF in the mPFCs of control, YTHDF1, YTHDF2, and YTHDF1+2 mice, by qPCR analysis (n=3). (F) mRNA levels of MFN, MFN2, DRP1, FIS1, OPA1, and MFF in N2a cells with overexpression of YTHDF1, YTHDF2, and YTHDF1+2, by qPCR analysis (n=3). ns not significant; *P<0.05; ***P<0.001 by ANOVA or Student's *t*-test; error bars denote the SEM.

# Figure S1

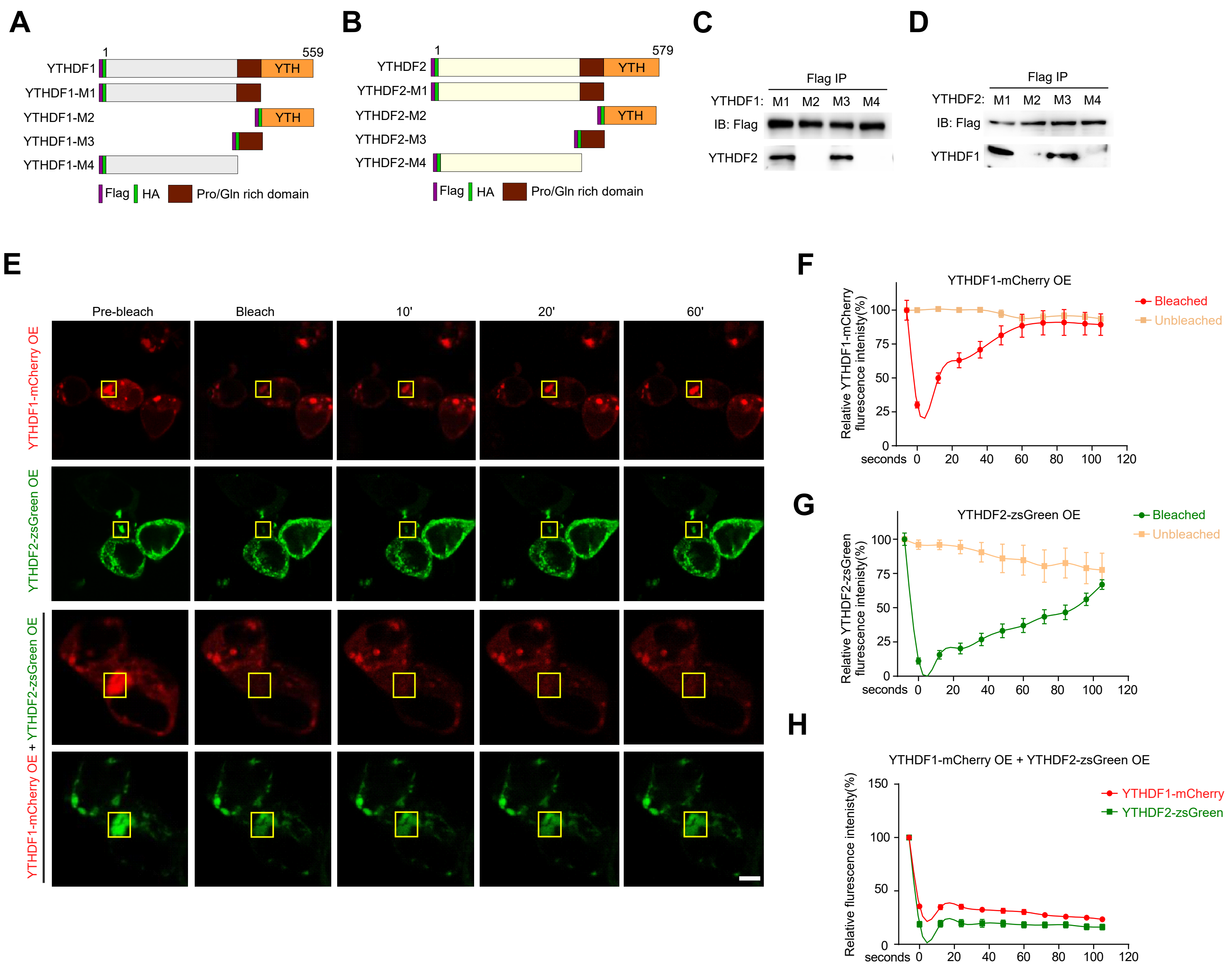
A
1
559
YTHDF1
YTH
YTHDF1-M1
YTHDF1-M2
YTH
YTHDF1-M3
YTHDF1-M4
Flag HA Pro/Gln rich domain
B
1
579
YTHDF2
YTH
YTHDF2-M1
YTHDF2-M2
YTH
YTHDF2-M3
YTHDF2-M4
Flag HA Pro/Gln rich domain
C
Flag IP
YTHDF1: M1 M2 M3 M4
IB: Flag
YTHDF2
D
Flag IP
YTHDF2: M1 M2 M3 M4
IB: Flag
YTHDF1
E
Pre-bleach
Bleach
10'
20'
60'
YTHDF1-mCherry OE
YTHDF2-zsGreen OE
YTHDF1-mCherry OE + YTHDF2-zsGreen OE
F
YTHDF1-mCherry OE
Bleached
Unbleached
Relative YTHDF1-mCherry flurescence intenisty(%)
seconds
G
YTHDF2-zsGreen OE
Bleached
Unbleached
Relative YTHDF2-zsGreen flurescence intenisty(%)
seconds
H
YTHDF1-mCherry OE + YTHDF2-zsGreen OE
YTHDF1-mCherry
YTHDF2-zsGreen
Relative flurescence intenisty(%)
seconds

# Figure S2

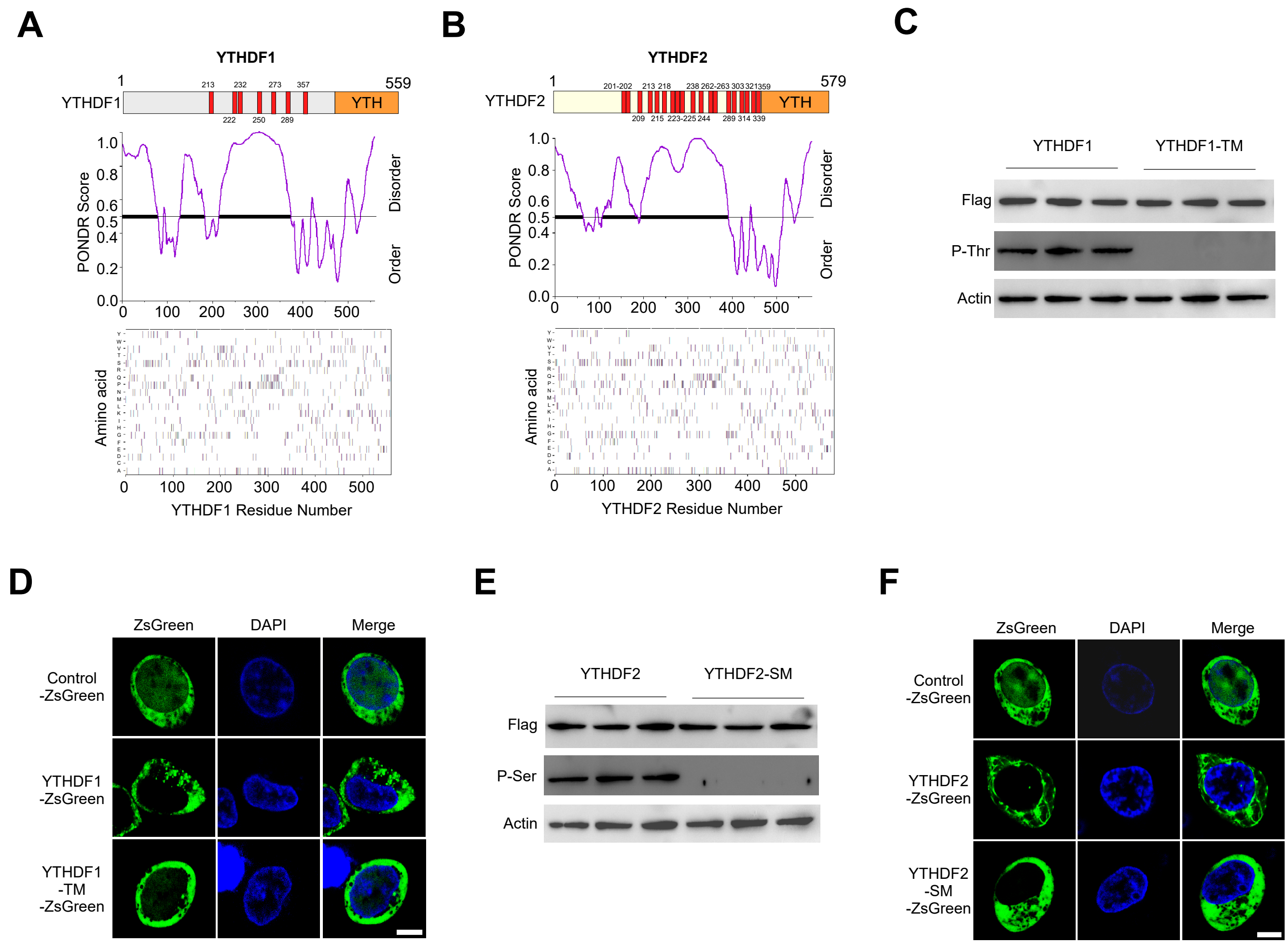

A
YTHDF1
1
559
YTHDF1
YTH
213
232
273
357
222
250
289
PONDR Score
1.0
0.8
0.6
0.5
0.4
0.2
0.0
0
100
200
300
400
500
Disorder
Order
Amino acid
YTHDF1 Residue Number
B
YTHDF2
1
579
YTHDF2
YTH
201-202
213
218
238
262-263
303
321
359
209
215
223-225
244
289
314
339
PONDR Score
Disorder
Order
Amino acid
YTHDF2 Residue Number
C
YTHDF1
YTHDF1-TM
Flag
P-Thr
Actin
D
ZsGreen
DAPI
Merge
Control -ZsGreen
YTHDF1 -ZsGreen
YTHDF1 -TM -ZsGreen
E
YTHDF2
YTHDF2-SM
Flag
P-Ser
Actin
F
ZsGreen
DAPI
Merge
Control -ZsGreen
YTHDF2 -ZsGreen
YTHDF2 -SM -ZsGreen

# Figure S3

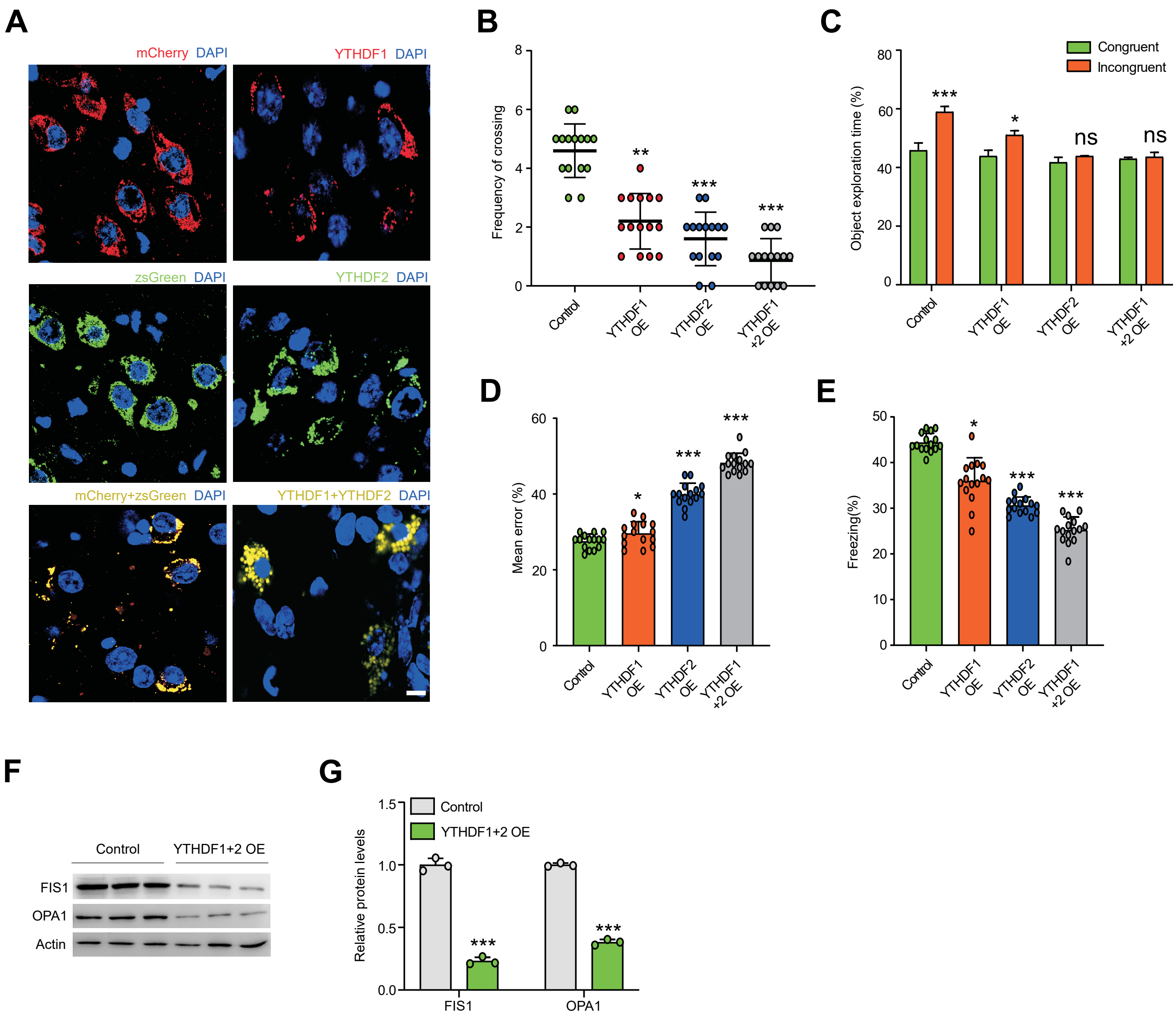
A
mCherry DAPI
YTHDF1 DAPI
zsGreen DAPI
YTHDF2 DAPI
mCherry+zsGreen DAPI
YTHDF1+YTHDF2 DAPI
B
Frequency of crossing
Control
YTHDF1 OE
YTHDF2 OE
YTHDF1 +2 OE
C
Object exploration time (%)
Congruent
Incongruent
ns
D
Mean error (%)
E
Freezing(%)
F
Control
YTHDF1+2 OE
FIS1
OPA1
Actin
G
Relative protein levels
Control
YTHDF1+2 OE
FIS1
OPA1

# Figure S4

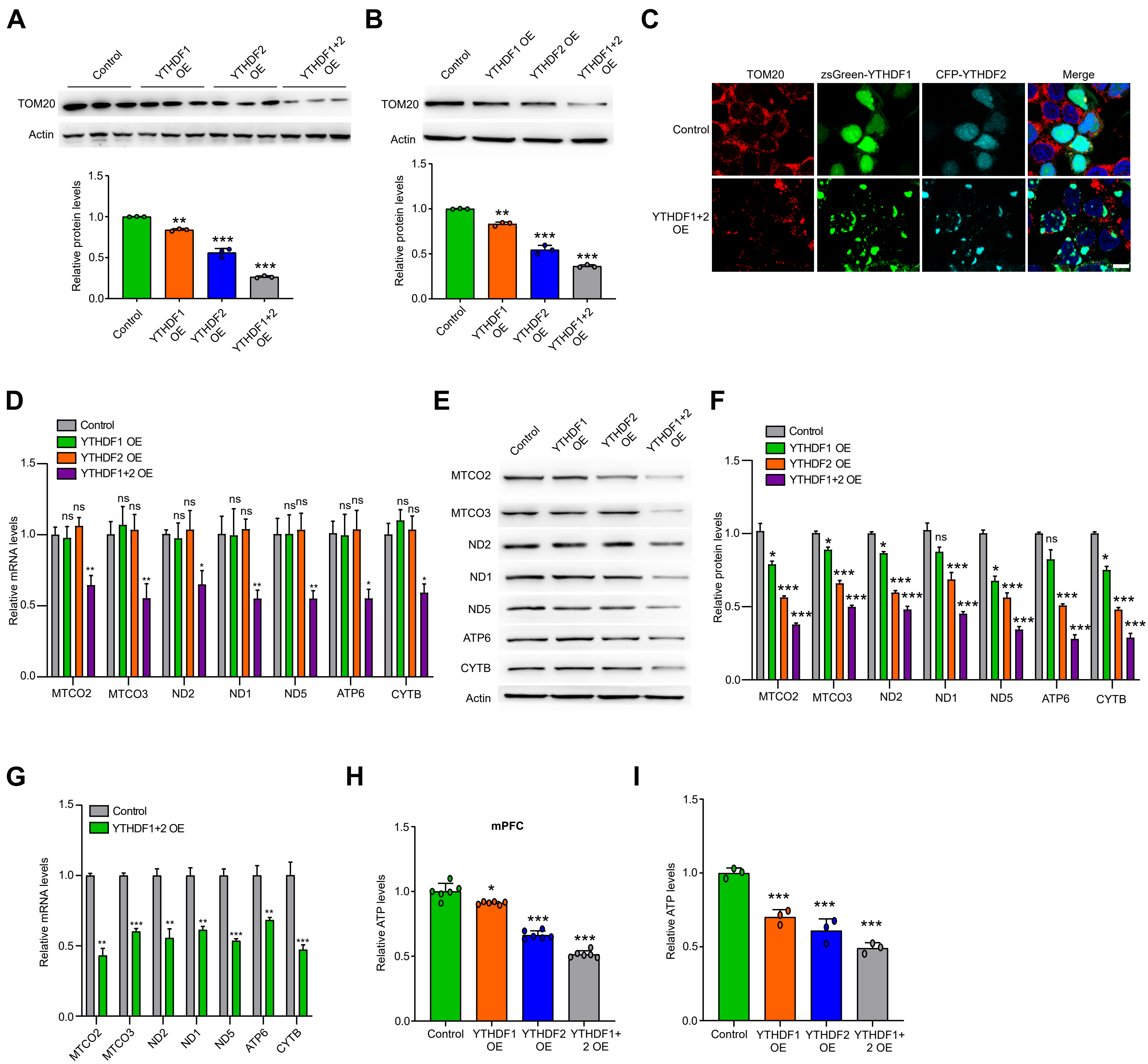

A
Control
YTHDF1 OE
YTHDF2 OE
YTHDF1+2 OE
TOM20
Actin
Relative protein levels
1.5
1.0
0.5
0.0
**
***
***
B
Control
YTHDF1 OE
YTHDF2 OE
YTHDF1+2 OE
TOM20
Actin
Relative protein levels
C
TOM20
zsGreen-YTHDF1
CFP-YTHDF2
Merge
Control
YTHDF1+2 OE
D
Control
YTHDF1 OE
YTHDF2 OE
YTHDF1+2 OE
Relative mRNA levels
ns
MTCO2
MTCO3
ND2
ND1
ND5
ATP6
CYTB
E
Control
YTHDF1 OE
YTHDF2 OE
YTHDF1+2 OE
MTCO2
MTCO3
ND2
ND1
ND5
ATP6
CYTB
Actin
F
Control
YTHDF1 OE
YTHDF2 OE
YTHDF1+2 OE
Relative protein levels
MTCO2
MTCO3
ND2
ND1
ND5
ATP6
CYTB
G
Control
YTHDF1+2 OE
Relative mRNA levels
MTCO2
MTCO3
ND2
ND1
ND5
ATP6
CYTB
H
mPFC
Relative ATP levels
Control
YTHDF1 OE
YTHDF2 OE
YTHDF1+2 OE
I
Relative ATP levels
Control
YTHDF1 OE
YTHDF2 OE
YTHDF1+2 OE

# Figure S5

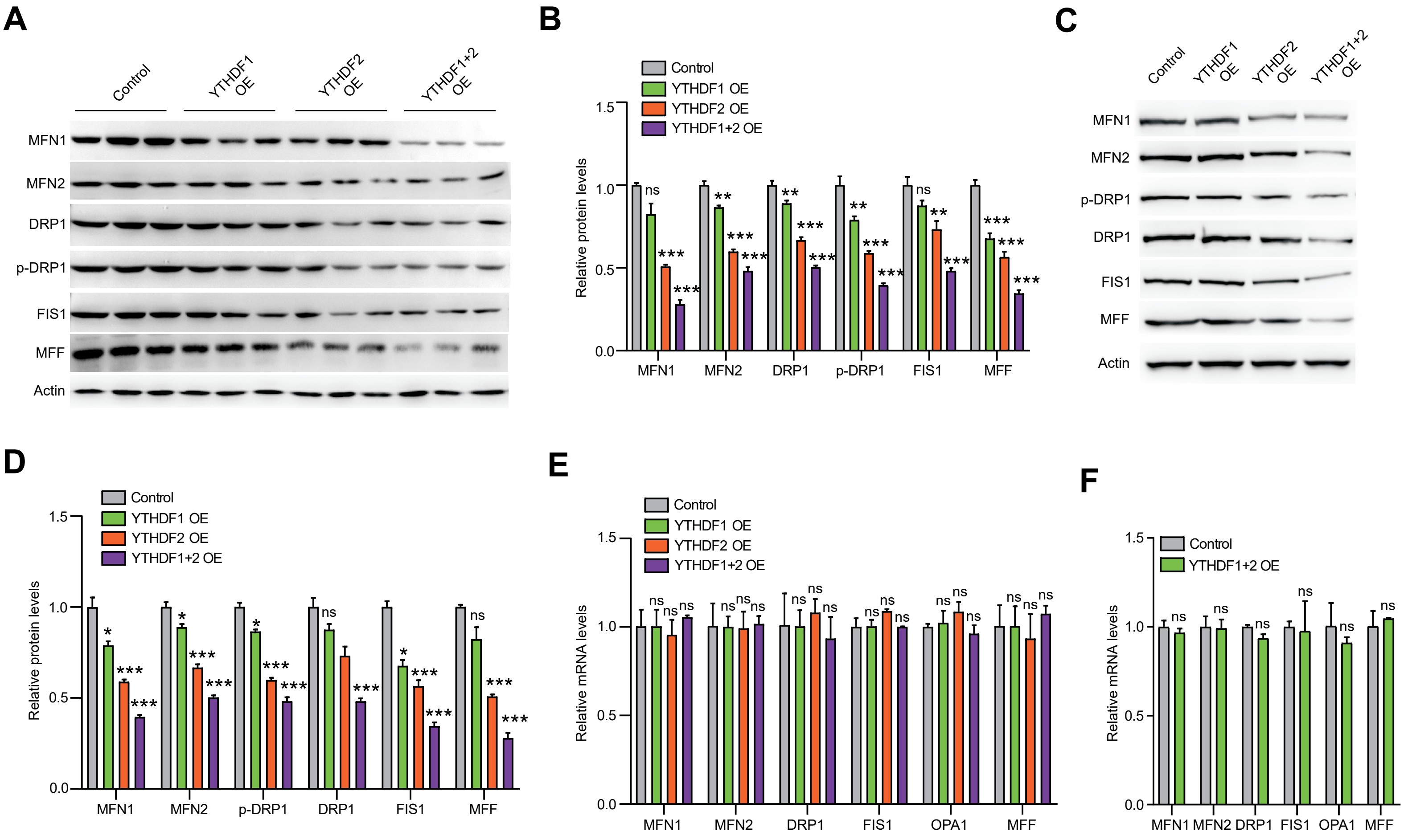

A
Control
YTHDF1 OE
YTHDF2 OE
YTHDF1+2 OE
MFN1
MFN2
DRP1
p-DRP1
FIS1
MFF
Actin
B
Control
YTHDF1 OE
YTHDF2 OE
YTHDF1+2 OE
Relative protein levels
1.5
1.0
0.5
0.0
MFN1
MFN2
DRP1
p-DRP1
FIS1
MFF
ns
**
***
C
Control
YTHDF1 OE
YTHDF2 OE
YTHDF1+2 OE
MFN1
MFN2
p-DRP1
DRP1
FIS1
MFF
Actin
D
Control
YTHDF1 OE
YTHDF2 OE
YTHDF1+2 OE
Relative protein levels
1.5
1.0
0.5
0.0
MFN1
MFN2
p-DRP1
DRP1
FIS1
MFF
*
***
ns
E
Control
YTHDF1 OE
YTHDF2 OE
YTHDF1+2 OE
Relative mRNA levels
1.5
1.0
0.5
0.0
MFN1
MFN2
DRP1
FIS1
OPA1
MFF
ns
F
Control
YTHDF1+2 OE
Relative mRNA levels
1.5
1.0
0.5
0.0
MFN1
MFN2
DRP1
FIS1
OPA1
MFF
ns
